# Discrepancy in the Location of Prostate Cancer Indicated on Biparametric Magnetic Resonance Imaging and Pathologically Diagnosed Using Surgical Specimens

**DOI:** 10.3390/curroncol31050216

**Published:** 2024-05-16

**Authors:** Masayuki Tomioka, Keita Nakane, Makoto Kawase, Koji Iinuma, Daiki Kato, Kota Kawase, Tomoki Taniguchi, Yuki Tobisawa, Fumiya Sugino, Tetsuro Kaga, Hiroki Kato, Masayuki Matsuo, Yusuke Kito, Chiemi Saigo, Natsuko Suzui, Takayasu Ito, Tatsuhiko Miyazaki, Tamotsu Takeuchi, Takuya Koie

**Affiliations:** 1 Department of Urology, Gifu University Graduate School of Medicine, Gifu 5011194, Japan; tomioka.masayuki.p7@s.gifu-u.ac.jp (M.T.); nakane.keita.k2@f.gifu-u.ac.jp (K.N.); kawase.makoto.g5@f.gifu-u.ac.jp (M.K.); iinuma.koji.s0@f.gifu-u.ac.jp (K.I.); kato.daiki.m2@f.gifu-u.ac.jp (D.K.); kawase.kota.b5@f.gifu-u.ac.jp (K.K.); taniguchi.tomoki.a8@f.gifu-u.ac.jp (T.T.); tobisawa.yuki.a7@f.gifu-u.ac.jp (Y.T.); 2 Department of Urology, Gifu Municipal Hospital, Gifu 5008513, Japan; f-sugino96tf@outlook.com; 3 Department of Radiology, Gifu University Graduate School of Medicine, Gifu 5011194, Japan; kaga.tetsuro.f7@f.gifu-u.ac.jp (T.K.); kato.hiroki.w4@f.gifu-u.ac.jp (H.K.); matsuo.masayuki.e0@f.gifu-u.ac.jp (M.M.); 4 Department of Pathology and Translational Research, Gifu University Graduate School of Medicine, Gifu 5011194, Japan; kito.yusuke.z1@f.gifu-u.ac.jp (Y.K.); saigoh.chiemi.n9@f.gifu-u.ac.jp (C.S.); takeuchi.tamotsu.p1@f.gifu-u.ac.jp (T.T.); 5 Department of Pathology, Gifu University Hospital, Gifu 5011194, Japan; suzui.natsuko.v0@f.gifu-u.ac.jp (N.S.); miyazaki.tatsuhiko.z0@f.gifu-u.ac.jp (T.M.); 6 Center for Clinical Training and Career Development, Gifu University Graduate School of Medicine, Gifu 5011194, Japan; ito.takayasu.v9@f.gifu-u.ac.jp

**Keywords:** prostate cancer, biparametric magnetic resonance imaging, prostate biopsy, tumor location, diagnostic discrepancy

## Abstract

Accurate diagnosis of the localization of prostate cancer (PCa) on magnetic resonance imaging (MRI) remains a challenge. We aimed to assess discrepancy between the location of PCa pathologically diagnosed using surgical specimens and lesions indicated as possible PCa by the Prostate Imaging Reporting and Data System on MRI. The primary endpoint was the concordance rate between the site of probable clinically significant PCa (csPCa) identified using biparametric MRI (bpMRI) and location of PCa in the surgical specimen obtained using robot-assisted total prostatectomy. Among 85 lesions identified in 30 patients; 42 (49.4%) were identified as possible PCa on MRI. The 85 PCa lesions were divided into positive and negative groups based on the bpMRI results. None of the patients had missed csPCa. Although the diagnostic accuracy of bpMRI was relatively high for PCas located in the middle of the prostate (*p* = 0.029), it was relatively low for PCa located at the base of the prostate, all of which were csPCas. Although current modalities can accurately diagnose PCa, the possibility that PCa is present with multiple lesions in the prostate should be considered, even if MRI does not detect PCa.

## 1. Introduction

Prostate cancer (PCa) is one of the most common cancers affecting men worldwide, accounting for approximately 30% of all malignant neoplasms [1]. Screening for serum prostate-specific antigen (PSA) levels, digital rectal examination, magnetic resonance imaging (MRI), and prostate biopsy guided by transrectal ultrasound (TRUS) in patients with abnormal results is the standard diagnostic method for clinically significant prostate cancer (csPCa) [2]. For patients with abnormal findings, MRI is performed and evaluated using the Prostate Imaging Reporting and Data System (PI-RADS) score version 2.1 [3]. Subsequently, TRUS-guided prostate biopsy is performed as a standard method for diagnosing csPCa [4]. Based on the National Comprehensive Cancer Network (NCCN) criteria, treatment options including surgery, radiation, and/or hormone therapy are proposed according to the risk classification of PCa and life expectancy of the patient [5].

Treatment strategies for PCa can be decided depending on clinical parameters such as PSA, PSA density, age, MRI findings, and grading of PCa by prostate biopsy. Among these, the diagnostic results of specimens obtained from TRUS-guided prostate biopsy play an important role in determining the risk classification [4,6]. However, TRUS-guided systematic biopsy (SB), which is the current standard method, results in misdiagnosis or misclassification in more than 50% of patients with PCa compared with pathology results from radical prostatectomy (RP) specimens [7]. Several guidelines recommend MRI-TRUS fusion-targeted biopsy (FUS-TB) using multiparametric MRI (mpMRI) in combination with T2-weighted (T2WI), diffusion-weighted (DWI), and dynamic contrast-enhanced images (DCE) [3,8,9]. In our previous studies, the diagnostic abilities for PCa were almost equivalent between mpMRI and biparametric MRI (bpMRI) using T2WI and DWI alone [10,11]. Prostate biopsies were performed using FUS-TB with bpMRI in combination with SB [12,13]. However, accurate diagnosis of the localization of all PCa on MRI remains a challenge [10]. In addition, the location, tumor volume, and/or grade of csPCa may not be accurately determined using only the previously described examination methods, and there is a risk of choosing a treatment method which does not treat the entire csPCa, such as focal therapy.

Therefore, in this study, we aimed to assess the discrepancy between the location of PCa pathologically diagnosed using surgical specimens removed via robot-assisted total prostatectomy (RARP) and that were indicated as possible PCa by the PI-RADS on MRI.

## 2. Materials and Methods

### 2.1. Enrolled Patients

This study was accepted by the Institutional Review Board of Gifu University (approval no.: 2022-192). Due to the retrospective nature of this study, written informed consent was not obtained from all the enrolled patients, and consent for the study was obtained through an opt-out procedure. Retrospective and observational studies in Japan require the disclosure of research information as well as existing materials, and this study was carried out based on the regulations and ethical guidelines of the Ethics Committee of Gifu University. The details of this study, which were provided in Japanese only, can be accessed at https://rinri.med.gifu-u.ac.jp/esct/publish_document.aspx?ID=2487 (accessed 27 February 2024).

This retrospective, single-center cohort study included patients with PCa who underwent RARP at Gifu University Hospital between September 2017 and September 2022. We collected the following data on patient characteristics and preoperative clinicopathological and laboratory parameters: age, height, weight, body mass index (BMI), preoperative serum PSA level, and prostate volume (PV). Eligible patients with PCa underwent computed tomography (CT), 99mTc-based bone scintigraphy, and bpMRI to confirm the absence of local invasion, lymph node metastasis, or distant metastasis before undergoing RARP. All patients underwent bpMRI before prostate biopsy, and prostate lesions were evaluated using PI-RADS version 2.1 [3]. When multiple lesions corresponding to the same PI-RADS category were present, the lesion with the largest diameter was considered the exponential lesion. The patients enrolled in this study underwent RARP without pelvic lymph node dissection according to a previously reported procedure [14,15].

### 2.2. bpMRI

Prior to prostate biopsy, the enrolled patients underwent bpMRI using a 3-Tesla clinical scanner equipped with a 32-channel phased-array receiver coil (Ingenia CX; Philips Healthcare, Best, The Netherlands). Using PI-RADS version 2.1, the MRI findings were described using T2WI and DWI scores. At least two radiologists with more than 5 years of extensive experience in diagnosing PCa evaluated the prostate based on bpMRI findings. In this study, the highest PI-RADS version 2.1 score obtained using bpMRI, regardless of the prostate region, was used for analysis.

### 2.3. Protocol for Prostate Biopsy

This study was conducted using data from prostate biopsies performed by more than 10 urologists. Briefly, TRUS-guided transperineal prostate biopsy was performed under spinal anesthesia using an 18G automated biopsy gun (PRIMECUT^®®^, Boston Scientific, Marlborough, MA, USA). Twelve tissue samples, including eight from the peripheral zone and four from the transitional zone, were collected for SB. The HI VISION Ascendus Sonography System (Hitachi Medical Corporation, Tokyo, Japan) was used for FUS-TB as the target biopsy. Two expert pathologists at our institution (T.M. and Tamotsu Takeuchi) evaluated the prostate biopsy specimens for the grade group (GG) based on the International Society of Urologic Pathology (ISUP) criteria [16].

### 2.4. Tumor Staging and the Assessment of Biopsy and Surgical Specimens

Tumor staging was evaluated in all cases according to the American Joint Committee on Cancer’s eighth edition of the Cancer Staging Manual [17].

All prostatectomy specimens were evaluated using whole-mount staining according to the ISUP guidelines [16]. The bladder neck sides of the prostate specimens were sectioned conically and then cut vertically. The remaining specimen was cut perpendicular to the urethral axis at 3–5 mm intervals. Sections (3 µm) were cut from the top surface of each section and stained with hematoxylin and eosin for subsequent light microscopic examination. The prostate biopsy specimens were evaluated using similar procedures. The size and location of the PCa were confirmed in each section. The GG was assessed on a per-patient basis rather than a per-lesion basis. In this study, we primarily focused on assessing the concordance between lesions identified in prostatectomy specimens and those detected on MRI. One urologist (M.T.) worked with the pathologists to identify the lesion site marked on the prepared slide and to assess whether the site was consistent with the lesion site noted on the bpMRI. We divided the prostate regions into ventral side, dorsal side, apex, middle, and base, rather than using the standardized radiological nomenclature that considers the zonal anatomy (transition zone, peripheral zone, and central zone) because of the distribution of PCa in various regions, which led to a more detailed classification. The GG of the biopsy and surgical specimens of the prostate were classified into the following five grades based on the International Society of Urologic Pathology (ISUP) 2014 guidelines [17]: GG1 (Gleason score [GS] ≤ 6), comprising only discrete individual well-formed glands; GG2 (GS 3 + 4 = 7), consisting of mainly well-formed glands with a small component of poorly formed/fused/cribriform glands; GG3 (GS 4 + 3 = 7), comprising mainly of poorly formed, fused, or cribriform gland ducts with few components of well-formed glands; GG4 (GS 8), consisting of poorly formed, fused, and cribriform glands only; and GG5 (GS9 or 10), exhibiting little or no gland formation with necrosis, whether or not poorly formed, fused, and cribriform glands are present. Positive lesions were defined as those showing prostate cancer on bpMRI, while negative lesions were those not showing prostate cancer on bpMRI. Lesions were assigned to the positive or negative group based on the presence or absence of prostate cancer on bpMRI, regardless of their PIRADS scores.

csPCa was defined as a grade ≥ 2 PCa based on the ISUP criteria and/or a maximum cancer core length ≥ 4 mm within at least one specimen obtained after MRI-TRUS fusion systematic or target biopsy [16,18]. Clinically insignificant prostate cancers (cisPCa) were defined as group 1 according to the 2014 ISUP guidelines [16,18].

### 2.5. Statistical Analysis

The primary endpoint was the concordance rate between the site of probable csPCa identified using bpMRI and location of PCa in the surgical specimen obtained using RARP. Patients with PCa that was consistent with the site where the bpMRI indicated PI-RADS ≥ 2 were defined as the positive group, and those with a diagnosis of PCa from a different site from where the lesion was indicated on bpMRI were defined as the negative group. In both groups, the number and location of csPCa lesions and GG were examined as secondary endpoints. Clinical and pathological factors were compared using JMP PRO version 16.2.0 (SAS Institute Inc., Cary, NC, USA). For comparisons between the positive and negative groups, clinical and pathological covariates were investigated using Pearson’s chi-square test. All *p*-values were two-sided, with *p* < 0.05 indicating statistical significance.

## 3. Results

### Selection of Eligible Patients and Patients’ Backgrounds

**Figure 1:**
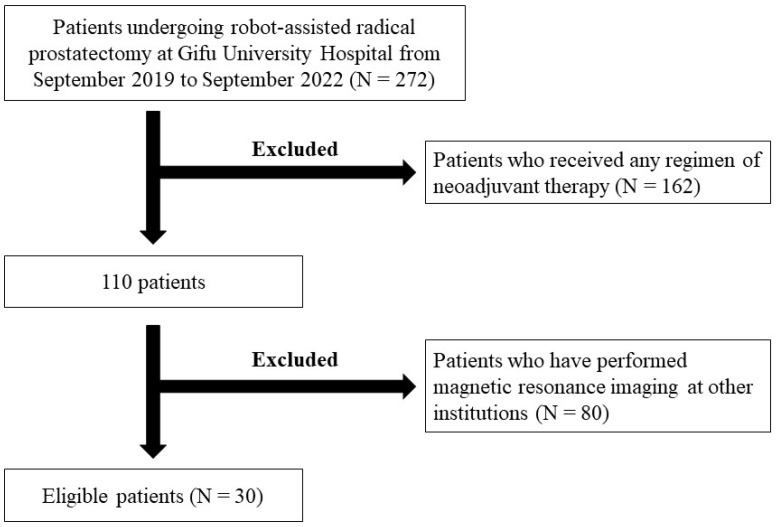
Patient selection eligibility criteria for this study.

Among 300 consecutive patients with PCa who underwent RARP at Gifu University Hospital between September 2017 and September 2022, we reviewed the clinical and pathological records of 272 patients with clinical T1/T2 PCa by NCCN risk classification. Patients who received any type of neoadjuvant therapy were excluded. Patients who underwent MRI at hospitals other than Gifu University were also excluded because of the possibility of inconsistencies in the PI-RADS evaluation. Finally, 85 lesions diagnosed as PCa based on surgical specimens from 30 patients were included in the study (Figure 1). Among them, 12 patients (40%) were enrolled in both groups.

Table 1 summarizes the clinical and pathological characteristics of the enrolled patients. Of 85 lesions identified in 30 patients, 42 (49.4%) were identified as possible PCa on MRI. Additionally, 7 patients (23.3%) had solitary PCa, whereas 23 (76.7%) had 2–6 tumors.

Table 2 shows the MRI findings and pathological evaluations of the positive and negative groups. None of the patients had missed csPCa because of PI-RADS ≥ 2 findings on bpMRI. However, 50.6% of patients had PCa in which the locations could not be identified on bpMRI. cisPCa was found in 6 out of 85 lesions, of which only 1 lesion (16.7%) could be identified on MRI.

The median cancer diameters on MRI and surgical specimens in enrolled patients were 10.0 mm (interquartile range [IQR], 6.9–13.1 mm) and 11.0 mm (IQR, 6.0–16.0 mm), respectively. Among the surgical specimens, 52 lesions had tumor diameters > 1 cm, of which 34 (40.0%) could be identified on MRI.

bpMRI was relatively accurate for diagnosing csPCa located in the middle of the prostate (*p* = 0.029). In contrast, when PCa was located at the base of the prostate, the diagnostic accuracy of bpMRI was relatively low, and all seven patients (100%) in the negative group had csPCa.

## 4. Discussion

mpMRI is a major instrument for optimizing prostate biopsies; for PCa with ISUP grade ≥ 2, its pooled sensitivity and specificity are 0.91 and 0.37, respectively, whereas for disease with an ISUP score ≥ 3, its pooled sensitivity and specificity are 0.95 and 0.35, respectively [9]. Although several guidelines recommend FUS-TB with mpMRI [3,8,9], the PCa detection rate with FUS-TB alone is limited to 25–50%, as reported in our previous studies [10,11,13,19,20]. SB may serve as a safety net in the absence of PCa on FUS-TB or for inaccurate MRI diagnoses [13]. Notably, the detection rate of csPCa is higher with combined FUS-TB and SB than with each biopsy method alone [21]. Günzel et al. reported an overall detection rate of 66% for PCa and 49% for csPCa with a combination of FUS-TB and SB, compared with a 22% detection rate for csPCa with SB alone [22]. In our previous study, the detection rate of PCa was 63.5% with SB alone, which increased to 75.0% when SB was combined with FUS-TB [10]. In contrast, Matsuoka et al. reported that csPCa was detected by SB outside the MRI target region, and in the PROMIS study, the negative predictive rate of PCa detection by MRI was 90% [23,24]. Of 736 patients with suspicious unilateral lesions on MRI, 145 (19.7%) had positive PCa detected by SB on the contralateral side [24]. For these reasons, MRI is less sensitive for identifying individual tumors in patients with GG 3–5 PCa, with approximately 22% being incorrectly identified and mislocated, and this proportion increases to 30% in patients with multiple foci [25]. Additionally, PCa may be missed during FUS-TB because of unexpected tissue deformation, a suboptimal needle trajectory due to inadequate contouring of the prostatic transection by MRI and/or TRUS, or inaccurate registration of the index lesion [26]. Heterogeneity in the quality of reporting owing to the involvement of various radiologists in prostate readings may also affect PCa detection rates [24]. Although the PI-RADS was evaluated in this study by two experts using bpMRI with 3-T MRI, csPCa was still missed in nearly half of the regions. The diagnostic ability in individual cases seems to be completely satisfactory because csPCa was noted in all cases; however, it may be difficult to identify all sites using MRI. The true clinical significance of missed tumor foci that cannot be detected on MRI remains controversial [27]. Recently, ligands for prostate-specific membrane antigens have been introduced into positron emission tomography for the diagnosis and management of PCa and have been useful in identifying the location of PCa and assessing tumor volume, which cannot be determined by MRI [28,29]. Future advances in diagnostic imaging may allow the precise identification of PCa location and tumor volume.

Discrepancies between MRI findings and prostate biopsy results, as well as between tumor sites in surgical specimens, are associated with the multifocal nature of PCa [27]. Of 736 patients with suspicious unilateral lesions on MRI, 145 (19.7%) had positive PCa detected by SB on the contralateral side [22]. Approximately 90% of cases with PCa location confirmed in surgical specimens have multifocal tumors [30]. In a study of surgical specimens, Noh et al. [27] reported that 81.2% of all patients had at least two PCa locations, 18.8% had one, 33.8% had two, and 33.8% had at least three PCa locations. An Australian study of 235 patients undergoing RP showed a sensitivity of 91% and a positive predictive value of 95% for identifying csPCa in patients with PI-RADS ≥ 3 on mpMRI [31]. In contrast, 9% of csPCa were determined to be normal on mpMRI, suggesting that the majority of cancers missed by mpMRI have a poorly formed cribriform architecture [31,32]. In the present study, approximately 90% of prostate biopsies and surgical specimens were GG ≤ 3, and the maximum diameter of the removed specimens was significantly shorter in the negative group than in the positive group (*p* < 0.001). Interestingly, mpMRI missed PCa located at the base of the prostate, and csPCa was detected in the surgical specimens of all patients. Several parameters, including tumor size, ISUP grade, location, and heterogeneous tumor morphology, may affect the visibility of PCa and accuracy of tumor volume assessment using MRI [33]. Although cisPCa was found in 6 of 85 lesions and only 1 out of these lesions was identified by MRI in this study, this suggests that the ability of MRI to detect PCa may be affected by tumor size and grade.

The validity of DCE for prostate MRI has been debated [34]. Some studies have demonstrated that DCE has a sensitivity of 60–74% [3,8,9] and improves the scoring of DWI [35], whereas others have suggested that DCE plays an insignificant role in detecting PCa [10,12,34,36]. DCE omission did not cause significant problems in the diagnostic accuracy or tumor detection rate of PCa, with an area under the curve (AUC) of 0.914 in the receiver operating characteristic curve analysis compared with an AUC of 0.917 for mpMRI, suggesting a high tumor detection ability in both methods [34]. A comparison of csPCa diagnostic ability between mpMRI and bpMRI using PI-RADS version 2.1 showed that mpMRI had significantly higher diagnostic sensitivity and bpMRI had significantly higher diagnostic specificity than their respective counterparts [36]. Furthermore, bpMRI showed a higher AUC for all lesions and for lesions in the peripheral regions than mpMRI [36]. In another study, although the sensitivity of bpMRI (59.1%) was lower than that of mpMRI (66.2%), the specificity of bpMRI (87.2%) was higher than that of mpMRI (84.6%) [10]. These results suggest that bpMRI has diagnostic capabilities comparable to those of mpMRI [36]. bpMRI without DCE-MRI requires less time than mpMRI and is more cost-effective for the patient [36]. Additionally, intravenous administration of gadolinium-based contrast agents increases not only the scan time but also the risk of developing contrast-agent-related adverse events, such as hypersensitivity reactions, renal systemic sclerosis in patients with chronic kidney disease, and gadolinium deposits in the brain [10]. Therefore, although mpMRI has a high diagnostic accuracy, PI-RADS using bpMRI may be a useful diagnostic tool when considering whether to perform a prostate biopsy.

This study has some limitations. First, the small number of patients enrolled and retrospective nature of the study may have introduced potential bias. Second, we did not directly compare bpMRI and mpMRI for PCa diagnosis using the PI-RADS. Therefore, the true effectiveness of bpMRI over mpMRI has not yet been confirmed. Third, it is necessary to consider that the excised specimens were cut at intervals of 3–5 mm, which may not accurately reflect the tumor location and volume. Fourth, not all PCas were identified in each patient in this study, and 40% of the patients were included in both groups. Therefore, a potential selection bias may be included. However, even when PCa cannot be identified by MRI, 88.4% of patients were diagnosed with csPCa, suggesting that this study may provide useful information. Finally, preoperative knowledge of the exact location and volume of PCa may aid in treatment selection; however, its direct impact on oncologic outcomes remains unclear.

## 5. Conclusions

Among 85 lesions identified in 30 patients, 42 (49.4%) were identified as possible PCa on MRI. Regarding the diagnosis of PCa using MRI, almost 100% of the lesions with positive bpMRI results indicated csPCa, whereas approximately 90% of the negative lesions also had csPCa. Although the diagnostic accuracy of bpMRI was relatively high for PCa located in the middle of the prostate, it was relatively low for PCa located at the base of the prostate, all of which were csPCa cases. As current modalities can accurately diagnose PCa, patients are unlikely to be at a disadvantage in terms of definitive treatment. Therefore, although the impact on oncological outcomes is unknown, the possibility that PCa is present with multiple lesions in the prostate should be considered, even if MRI cannot confirm PCa. A prospective study with a large number of cases is needed to compare the distribution of PCa with the results obtained using various imaging modalities, including MRI.

## Figures and Tables

**Table 1:** Patient background of all enrolled patients.

Variables	Patients (N = 30)
Age, years (median, IQR)	73 (68–75)
Body mass index, kg/m^2^ (median, IQR)	23.1 (21.2–24.8)
Initial prostate-specific antigen level, ng/mL (median, IQR)	6.36 (4.73–10.38)
Prostate volume, mL (median, IQR)	31.0 (24.3–43.6)
Clinical T stage (number, %)	
1c	4 (13.3)
2a	20 (66.7)
2b	1 (3.3)
2c	5 (16.7)
Biopsy grade group according to ISUP criteria (number, %)	
1	7 (23.3)
2	14 (46.7)
3	8 (26.7)
4	0 (0.0)
5	1 (3.3)
NCCN risk classification (number, %)	
Very Low	1 (3.3)
Low	5 (16.7)
Intermediate	23 (76.7)
High	1 (3.3)
Pathological T stage (number, %)	
2a	7 (23.3)
2b	2 (6.7)
2c	16 (53.3)
3a	3 (10.0)
3b	2 (6.7)
Surgical specimens grade group according to ISUP criteria (number, %)	
1	12 (14.1)
2	45 (52.9)
3	22 (25.9%)
4	0 (0.0%)
5	6 (7.1%)
Follow-up period, months (median, IQR)	25 (12–30)

IQR, interquartile range; ISUP, the International Society of Urologic Pathology; NCCN, the National Comprehensive Cancer Network.

**Table 2:** Magnetic resonance imaging (MRI) findings and pathological evaluation in the positive (lesions showing prostate cancer on biparametric MRI) and negative (no lesions showing prostate cancer on biparametric MRI) groups.

Variables	Positive Group (n = 42)	Negative Group (n = 43)	*p*-Value
MRI findings using PI-RADS score version 2.1 (number, %)			<0.001
1	1 (2.4)	20 (46.5)	
2	1 (2.4)	9 (20.9)	
3	12 (28.6)	8 (18.6)	
4	26 (61.9)	6 (14.0)	
5	2 (4.8)	0 (0.0)	
Biopsy grade group according to ISUP criteria (number, %)			0.288
1	11 (26.2)	10 (23.3)	
2	20 (47.6)	18 (41.9)	
3	11 (26.2)	11 (25.6)	
4	0 (0.0)	0 (0.0)	
5	0 (0.0)	4 (9.3)	
Surgical specimen grade group according to ISUP criteria (number, %)			0.112
1	6 (14.3)	6 (14.0)	
2	20 (47.6)	25 (58.1)	
3	15 (35.7)	7 (16.3)	
4	0 (0.0)	0 (0.0)	
5	1 (2.4)	5 (11.6)	
Maximum cancer diameter on MRI lesions, mm (median, IQR)	10.0 (6.50–13.0)	8.0 (8.0–8.0)	0.662
Maximum cancer diameter on surgical specimens, mm (median, IQR)	13.5 (10.0–19.5)	6.0 (4.0–12.5)	<0.001
Localization of prostate cancer (number, %)			
Ventral side of the prostate	29 (69.0)	23 (53.5)	0.327
Dorsal side of the prostate	13 (31.0)	20 (46.5)	0.139
Apex of the prostate gland	10 (23.8)	12 (27.9)	0.763
Middle of the prostate gland	32 (76.2)	24 (55.8)	0.186
Base of the prostate gland	0 (0.0)	7 (16.3)	<0.001
Clinically significant prostate cancer (%)	41 (97.6)	38 (88.4)	0.202
Ventral side of the prostate	29 (100)	21 (91.3)	0.191
Dorsal side of the prostate	12 (92.3)	17 (85.0)	>0.999
Apex of the prostate gland	9 (90.0)	11 (91.7)	>0.999
Middle of the prostate gland	32 (100)	20 (83.3)	0.029
Base of the prostate gland	0	7 (100)	<0.001

MRI, magnetic resonance imaging; PI-RADS, the Prostate Imaging Reporting and Data System; ISUP, the International Society of Urologic Pathology; IQR, interquartile range.

## Data Availability

All data and materials are made available in this paper.
